# Breast Cancer with a Newly Diagnosed Variant in the *PTEN* Gene: A Case Report

**DOI:** 10.70352/scrj.cr.24-0082

**Published:** 2025-01-31

**Authors:** Yuka Maeda, Tatsuhiko Ikeda, Ayana Sato, Akiko Matsumoto, Hiromitsu Jinno

**Affiliations:** Department of Surgery, School of Medicine, Teikyo University, Tokyo, Japan

**Keywords:** hereditary breast cancer, Cowden syndrome, PTEN

## Abstract

**INTRODUCTION:**

The phosphatase and tensin homolog hamartoma tumor syndrome (PHTS) refers to a spectrum of disorders caused by variants of the phosphatase and tensin homolog (*PTEN*) gene, including Cowden syndrome (CS), Bannayan–Riley–Ruvalcaba syndrome, adult Lhermitte–Duclos disease, and autism spectrum disorders associated with macrocephaly. PHTS is characterized by hamartomas in multiple organs and is associated with an increased risk of developing malignant tumors including, breast, thyroid, endometrial, colorectal, and kidney tumors. Breast cancer is the most common malignancy associated with PHTS.

**CASE PRESENTATION:**

We describe the case of a 44-year-old female patient with invasive ductal carcinoma of the right breast. Cobblestone papillomatosis was present in the gingiva. She had a medical history of bilateral adenomatous goiters for 10 years. Her mother had been diagnosed with breast cancer, thyroid and tongue tumors, gastric polyps, hepatic hemangioma, and collagen disease. Additionally, the patient’s maternal grandmother had a history of colon cancer. Based on the patient’s family history and physical findings, CS was suspected, and direct DNA sequencing analysis revealed a haplotype c.634del mutation in exon 7 of the *PTEN* gene. Although there is no clear evidence supporting risk-reducing surgery for PHTS, a right nipple-sparing mastectomy, sentinel lymph node biopsy, and tissue expander reconstruction were performed.

**CONCLUSIONS:**

We report a case of breast cancer with a newly diagnosed c.634del mutation in the *PTEN* gene. We also reviewed the current literature on *PTEN* genetic variants and breast cancer subtypes.

## Abbreviations

NCCN
: National Comprehensive Cancer Network
PHTS
: PTEN hamartoma tumor syndrome
PTEN
: phosphatase and tensin homolog
CS
: Cowden syndrome
LDD
: Lhermitte–Duclos disease

## INTRODUCTION

The phosphatase and tensin homolog hamartoma tumor syndrome (PHTS) refers to a spectrum of disorders caused by variants of the phosphatase and tensin homolog (*PTEN*) gene, including Cowden syndrome (CS), Bannayan–Riley–Ruvalcaba syndrome, adult Lhermitte–Duclos disease (LDD), and autism spectrum disorders associated with macrocephaly. CS is characterized by multiple hamartomas and carries a high risk of both benign and malignant tumors of the breast, thyroid, endometrium, colorectum, and kidneys.^1)^ The most common malignancy associated with PHTS is breast cancer, with a reported lifetime risk of 85%.^2)^ Here, we report a case of breast cancer with a novel mutation in the *PTEN* gene.

## CASE PRESENTATION

**Fig. 1:**
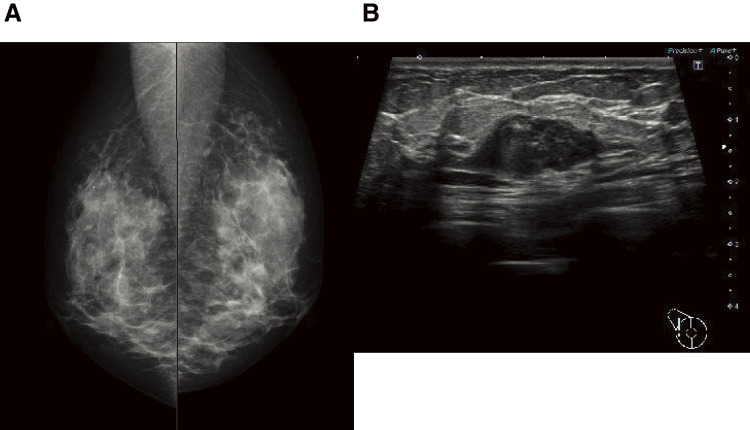
(**A**) Mammography showed no tumor or calcification in her bilateral breast. (**B**) Ultrasonography showed a circumscribed, irregular hypoechoic mass in her right breast.

**Fig. 2:**
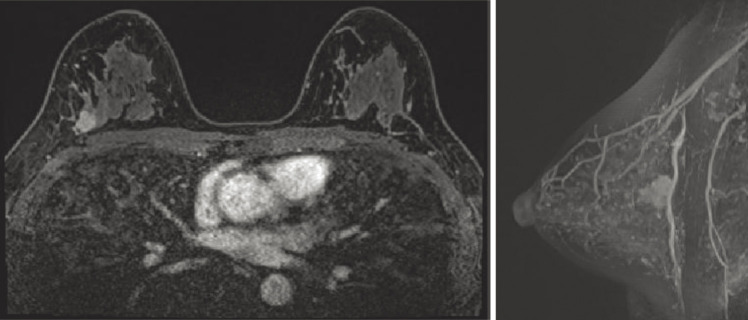
Contrast-enhanced magnetic resonance imaging showed an enhanced irregular mass in the right breast.

**Fig. 3:**
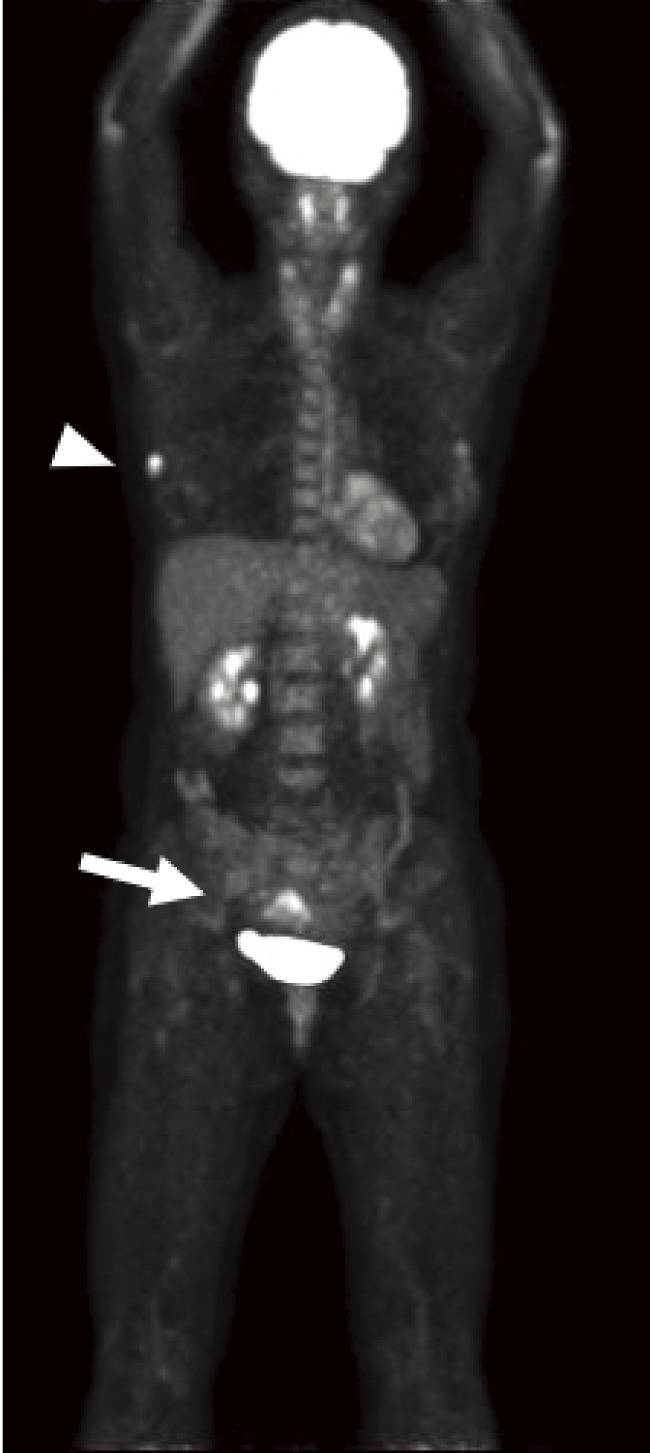
Fluorodeoxyglucose-positron emission tomography showed abnormal resorption in her right breast (arrowhead) and uterus (arrow).

**Fig. 4:**
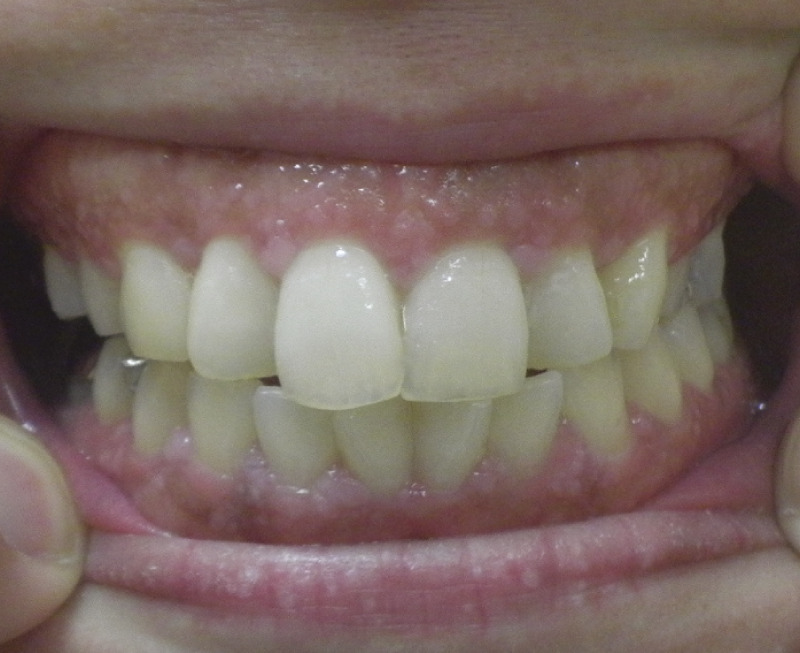
Papillomatosis in a cobblestone was present on her gingivae.

**Fig. 5:**
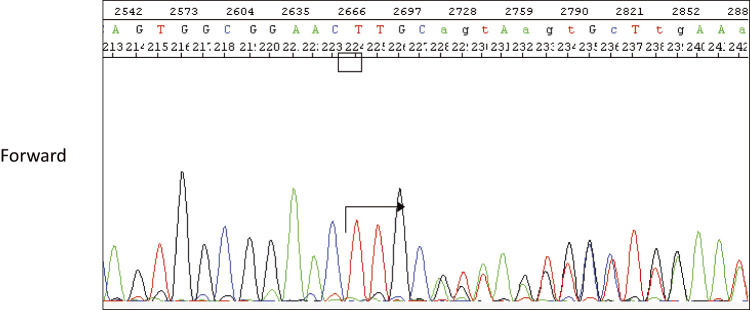
A direct DNA sequencing analysis showed a haplotype c.634del mutation of the PTEN gene.

A 44-year-old woman was referred to our hospital because of a right breast tumor identified during a medical checkup. Mammography revealed no tumor or calcification in either breast. However, ultrasonography revealed an irregular hypoechoic mass (22 × 19 × 9 mm) in the right breast (**Fig. 1**). Dynamic contrast-enhanced magnetic resonance imaging showed an enhanced, irregular mass in the right breast (**Fig. 2**). Fluorodeoxyglucose positron emission tomography revealed abnormal resorption in the right breast and uterus (**Fig. 3**). Abnormal resorption in the uterus was diagnosed as a uterine myoma on computed tomography. A core needle biopsy revealed an invasive ductal carcinoma that was estrogen receptor (ER) positive, progesterone receptor (PR) positive, and human epidermal receptor 2 (HER2) negative. She had a medical history of bilateral adenomatous goiters for 10 years. Cobblestone papillomatosis was present in the gingiva (**Fig. 4**). Her mother had been diagnosed with breast cancer, thyroid and tongue tumors, gastric polyps, hepatic hemangioma, and collagen disease. Additionally, her maternal grandmother had been diagnosed with colon cancer. Owing to her family history and physical findings, CS was suspected. The diagnosis of CS was based on the criteria of the National Comprehensive Cancer Network (NCCN).^3)^ In this case, breast cancer and oral papilloma met the major criteria, and thyroid structural lesions met the minor criteria; however, the diagnostic criteria for CS were not met. Despite this, due to the strong suspicion of CS based on her mother’s medical history, genetic testing was performed prior to surgery. From an ethical perspective, professional genetic counseling was provided to the patient and her family, and informed consent for genetic testing was obtained. A direct DNA sequencing analysis was performed, and a haplotype c.634del mutation in exon 7 of the *PTEN* gene was detected (**Fig. 5**). This mutation resulted in a p.Asn212Ilefs*9 substitution. Based on the results of the genetic test, she was diagnosed with PHTS.

**Fig. 6:**
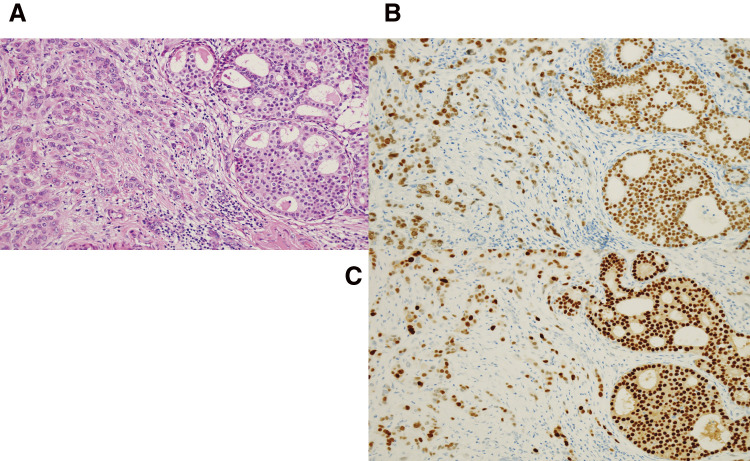
Histopathological examination of the right breast tumor ×200. (**A**) Hematoxylin and eosin staining shows an invasive ductal carcinoma with histological grade II. Immunohistochemistry revealed ER positive (**B**) and PR positive (**C**).

Although there is no clear evidence for risk-reducing surgery in PHTS, a nipple-sparing mastectomy, sentinel lymph node biopsy, and tissue expander reconstruction were performed. Histopathological examination confirmed an invasive ductal carcinoma measuring 1.8 × 1.0 cm with no lymph node metastasis (**Fig. 6**). The histological grade was II. Immunohistochemistry revealed ER- and PR-positive staining, HER-2-negative status, and Ki-67-positive staining of 15%. Aromatase inhibitor therapy was started as adjuvant therapy. Five months after breast cancer surgery, the patient underwent a laparoscopic total hysterectomy and bilateral salpingo-oophorectomy, and the histopathological diagnosis was atypical endometrial hyperplasia.

## DISCUSSION

The *PTEN* gene has two main domains: the N-terminal region, which includes the phosphatase domain, containing the *PTEN* active site in exon 5, and the C-terminal region, which includes the C2 domain, that binds to phospholipids and positions the catalytic domain on the membrane.^4)^ According to a study investigating cancer risk and genotype–phenotype correlations in 22 Japanese patients with CS, 12 had N-terminal-*PTEN* variants (11 in the phosphatase domain) and 10 had C-terminal-*PTEN* variants (C2 domain). Cancer was found in 33.3% (4/12) of the patients in the N-terminal region group (27.3% [3/11] in the phosphatase domain) and 80.0% (8/10) of those in the C2 domain group. The incidence of cancer in the C2 domain group was significantly higher than that in the N-terminal region (p = 0.038) and phosphatase domain (p = 0.023) groups. Breast cancer was found in 100% (7/7) of female patients in the C2 domain group and 50.0% (4/8) of those in the N-terminal group (42.9% [3/7] of those in the phosphatase domain group). The incidence of breast cancer in the C2 domain group was marginally higher than that in the N-terminal region group (p = 0.051).^5)^

Pathogenic variants of the *PTEN* gene may include large deletions, small intragenic deletions/insertions and missense, nonsense, and splice site variants, and these have been described in all nine exons of the gene.^2,6–8)^ Unlike other genes, virtually all germline *PTEN* missense mutations in the coding region are believed to be pathogenic.^6,9)^ Furthermore, no clear genotype–phenotype correlation has been found in PHTS, although several studies have examined the correlation between CS phenotypes and *PTEN* gene variant sites.^2,4,10,11)^

The c.634del mutation in the C2 domain has not previously been reported in PHTS. Germline *PTEN*-related breast cancer is characterized by a high incidence of hormone receptor-positive cases, the progression tends to be slow, and the prognosis is not poor.^5)^

On the other hand, somatic *PTEN* mutation has been reported to be associated with triple-negative breast cancers.^12)^ The literature describing both PTEN variants and breast cancer subtypes is presented in **Table 1**. Hormone receptor-positive cases were the most common, including the present case (8 out of 12 cases), whereas 3 and 1 cases were triple negative and HER2 positive, respectively.^13–22)^

**Table 1:** Expression of hormone receptor of breast cancer and PTEN mutations in PHTS

Age	Sex	ER	PgR	HER2	Pathology	Bilateral	Exon	Nucleotide change	Variant type	Reference
32	F	+	+	–	IDC	+	1	c.71A>T	Missense	Pradella et al., 2014^13)^
33	F	+	+	–	IDC	+	5	p.R130*(c.388C>T)	Nonsense	Nara et al., 2017^14)^
29	F	+	NA	–	IDC	+	5	p.R130Q(c.389G>A)	Missense	Chandhanayingyong et al., 2015^15)^
57	F	–	–	–	Mucinous	–	7	c.697C>T	Nonsense	Gosein et al., 2016^16)^
34	F	+	NA	–	IDC	–	7	c.698_701delGACCinsAA	NA	Walsh et al., 2011^17)^
29	F	–	–	–	IDC	+	7	c.723dupT	Nonsense	Won et al., 2019^18)^
35	F	+	+	–	DCIS	–	7	c.723dupT	Nonsense	Nara et al., 2017^14)^
41	M	+	NA	NA	IDC	–	7	c.802delG	Splicing	Fackenthal et al., 2001^19)^
35	F	–	–	–	IDC	–	8	c.823_840del.18	Nonsense	Sueta et al., 2022^20)^
44	F	+	+	–	IDC	+	9	c.1027-2A>G	Splicing	Peiró et al., 2010^21)^
38	F	–	–	+	IDC	–	NA	Mutation	NA	Sabaté et al., 2006^22)^
44	F	+	+	–	IDC	–	7	c.634del	Frameshift	This case

NA, not assessed; IDC, invasive ductal carcinoma; DCIS, ductal carcinoma in situ.

PHTS is a rare autosomal dominant hereditary disorder caused by germline variants of the *PTEN* gene located on chromosome 10q23.^6,23,24)^ *PTEN* participates in the negative regulation of the phosphoinositide 3-kinase-protein kinase B and mammalian target of rapamycin signaling pathways, controlling cell proliferation and cell cycle progression and promoting apoptosis.^25)^ Thus, the loss-of-function mutation of *PTEN* correlates with the development of various human cancers. In recent studies, it has been found that target the cellular alterations caused by *PTEN* pathogenic variants.^26)^ Pharmacologic mTOR inhibitors (like everolimus and sirolimus) are being studied for their potential to reduce oncologic risk. Additionally, there is a hypothesis that poly (ADP-ribose) polymerase (PARP) inhibitors may also be effective for *PTEN* pathogenic variant carriers, similar to their use in *BRCA*-related breast cancers. However, more research is needed to effectively translate these strategies into prevention and treatment options. The cumulative lifetime risk of any cancer in patients with CS is 89%, with morbidities including 85% for breast cancer, 32% for LDD, 21% for thyroid cancer, 19% for endometrial cancer, 16% for colorectal cancer, and 15% for renal cancer.^2,27)^ Nevertheless, the most typical features are specific mucocutaneous lesions, including trichilemmomas, acral keratoses, and oral papillomatous papules, which occur in 90%–100% of patients with CS.

Cancer surveillance should be performed for individuals with positive findings on genetic testing and those with negative findings on genetic testing but meeting the clinical diagnostic criteria. The NCCN guideline recommends breast self-examination beginning at the age of 18 years, annual clinical breast examinations starting at the age of 25 years, and mammography and breast magnetic resonance imaging (MRI) at 30–35 years of age (or 5–10 years before a family’s earliest known breast cancer diagnosis).^28)^ Given the 85% risk of developing breast cancer, the possibility of risk-reducing mastectomy should be considered. Counseling should include discussions on the degree of protection, reconstruction options, and associated risks. While options like nipple-sparing mastectomy show promise based on *BRCA* data, the effectiveness of chemoprevention in *PTEN* pathogenic variant carriers remains unclear. Overall, further research is essential to improve outcomes and clarify treatment strategies for this population.^26)^ Early detection and surveillance of *PTEN*-related hereditary cancers is done with established imaging modalities such as ultrasound and MRI (**Table 2**). In the present case, the patient should undergo future surveillance of the left breast, thyroid, kidneys, and colon including annual MRI and mammography for breast evaluation, annual ultrasound for thyroid assessment, ultrasound every 1–2 years for renal cancer surveillance, and a colonoscopy every 5 years for colorectal cancer screening.

**Table 2:** Guideline summary: cancer surveillance protocol for individuals with PTEN hamartoma tumor syndrome

	Surveillance	Interval	From age
Breast cancer	Breast self-examination	Every 1 year	18
	MRI	Every 1 year	30
	Mammography	Every 1 year	30–35
	Risk-reducing surgery offered	–	–
Thyroid cancer	Ultrasound	Every 1 year	Age at diagnosis
Renal cancer	Ultrasound	Every 1–2 years	40
Colorectal cancer	Baseline colonoscopy	Every 5 years	35–40
Melanoma	Baseline skin examination	–	30
Endometrial cancer	The presence or absence of abnormal bleeding/endometrial biopsy^※^	–	–

^※^This should include endometrial biopsy every 1–2 years if abnormal uterine bleeding or postmenopausal bleeding occurs.

## CONCLUSION

Herein, we report a case of breast cancer with a newly diagnosed c.634del mutation in the *PTEN* gene. Because the patient was diagnosed with PHTS before breast cancer surgery, we were able to discuss the breast surgery procedure resulting in her undergoing a right nipple-sparing mastectomy. We also reviewed the current literature on *PTEN* genetic variants and breast cancer subtypes.

## ACKNOWLEDGMENTS

The authors thank all those who contributed to this report.

## DECLARATIONS

### Funding

This study did not receive any specific grants from funding agencies in the public, commercial, or non-profit sectors.

### Authors’ contributions

YM and HJ performed the surgical procedures.

All the authors have read and approved the final version of the manuscript.

### Availability of data and materials

The data are not available for public access due to patient privacy concerns but are available from the corresponding author upon reasonable request.

### Ethics approval and consent to participate

Ethics approval: All procedures involving human participants were performed in accordance with the ethical standards of the Institutional Review Board of Teikyo University Hospital and the 1964 Helsinki Declaration and its later amendments.

### Consent for publication

Informed consent was obtained from the patient for the publication of this report.

### Competing interests

The authors declare that they have no competing interests.

## References

[1] Pilarski R Burt R Kohlman W Cowden syndrome and the PTEN hamartoma tumor syndrome: systematic review and revised diagnostic criteria. J Natl Cancer Inst 2013; 105: 1607–16. 24136893 10.1093/jnci/djt277

[2] Tan MH Mester JL Ngeow J Lifetime cancer risks in individuals with germline PTEN mutations. Clin Cancer Res 2012; 18: 400–7. 22252256 10.1158/1078-0432.CCR-11-2283 PMC3261579

[3] NCCN guidelines. Genetic/familial high-risk assessment: breast and ovarian, version 3. 2019, 2019. https://www2.tri-kobe.org/nccn/guideline/gynecological/english/genetic_familial.pdf#search=%27NCCN+guidline%2C+hereditary+and+familial+breast+and+ovarian%27. 10.6004/jnccn.2017.0003 28040716

[4] Bubien V Bonnet F Brouste V High cumulative risks of cancer in patients with PTEN hamartoma tumour syndrome. J Med Genet 2013; 50: 255–63. 23335809 10.1136/jmedgenet-2012-101339

[5] Teramae S Muguruma N Okamoto K Cancer risk and genotype-phenotype correlation in Japanese patients with Cowden syndrome. Int J Clin Oncol 2022; 27: 639–47. 35106660 10.1007/s10147-022-02116-w

[6] Tan MH Mester J Peterson C A clinical scoring system for selection of patients for PTEN mutation testing is proposed on the basis of a prospective study of 3042 probands. Am J Hum Genet 2011; 88: 42–56. 21194675 10.1016/j.ajhg.2010.11.013 PMC3014373

[7] Ngeow J Stanuch K Mester JL Second malignant neoplasms in patients with Cowden syndrome with underlying germline PTEN mutations. J Clin Oncol 2014; 32: 1818–24. 24778394 10.1200/JCO.2013.53.6656 PMC4039869

[8] Mester J Eng C. When overgrowth bumps into cancer: the PTEN-opathies. Am J Med Genet C Semin Med Genet 2013; 163: 114–21. 10.1002/ajmg.c.31364 23613428

[9] Zbuk KM Eng C. Cancer phenomics: RET and PTEN as illustrative models. Nat Rev Cancer 2007; 7: 35–45. 17167516 10.1038/nrc2037

[10] Nieuwenhuis MH Kets CM Murphy-Ryan M Cancer risk and genotype-phenotype correlations in PTEN hamartoma tumor syndrome. Fam Cancer 2014; 13: 57–63. 23934601 10.1007/s10689-013-9674-3

[11] Leslie NR Longy M. Inherited PTEN mutations and the prediction of phenotype. Semin Cell Dev Biol 2016; 52: 30–8. 26827793 10.1016/j.semcdb.2016.01.030

[12] Cancer Genome Atlas Network. Comprehensive molecular portraits of human breast tumours. Nature 2012; 490: 61–70. 23000897 10.1038/nature11412 PMC3465532

[13] Pradella LM Evangelisti C Ligorio C A novel deleterious PTEN mutation in a patient with early-onset bilateral breast cancer. BMC Cancer 2014; 14: 70. 24498881 10.1186/1471-2407-14-70 PMC3922036

[14] Nara M Ishida N Hagio K Two cases of breast cancer in juvenile patients in their thirties with pten hamartoma tumor syndrome. J Jpn Surg Assoc 2017; 78: 2624–8. (in Japanese)

[15] Chandhanayingyong MC Bernthal NM Ungarreevittaya P Ewing sarcoma in a patient with Cowden syndrome. J Natl Compr Canc Netw 2015; 13: 1310–4. 26553762 10.6004/jnccn.2015.0161

[16] Gosein MA Narinesingh D Nixon CA-AC Multi-organ benign and malignant tumors: recognizing Cowden syndrome: a case report and review of the literature. BMC Res Notes 2016; 9: 388. 27488391 10.1186/s13104-016-2195-z PMC4973052

[17] Walsh S Carter M Tubridy N Lhermitte-Duclos and Cowden diseases: breast cancer as an unusual initial presentation of these overlapping conditions. BMJ Case Rep 2011; 2011: bcr0820114730. 10.1136/bcr.08.2011.4730 PMC3207787 22675060

[18] Won HS Chang ED Na SJ PTEN mutation identified in patient diagnosed with simultaneous multiple cancers. Cancer Res Treat 2019; 51: 402–7. 29510612 10.4143/crt.2017.579 PMC6333971

[19] Fackenthal JD Marsh DJ Richardson AL Male breast cancer in Cowden syndrome patients with germline PTEN mutations. J Med Genet 2001; 38: 159–64. 11238682 10.1136/jmg.38.3.159 PMC1734834

[20] Sueta A Takeno M Goto-Yamaguchi L A progressive and refractory case of breast cancer with Cowden syndrome. World J Surg Oncol 2022; 20: 279. 36057718 10.1186/s12957-022-02745-5 PMC9440557

[21] Peiró G Adrover E Guijarro J Synchronous bilateral breast carcinoma in a patient with Cowden syndrome: a case report with morphologic, immunohistochemical and genetic analysis. Breast J 2010; 16: 77–81. 19968660 10.1111/j.1524-4741.2009.00846.x

[22] Sabaté JM Gómez A Torrubia S Evaluation of breast involvement in relation to Cowden syndrome: a radiological and clinicopathological study of patients with PTEN germ-line mutations. Eur Radiol 2006; 16: 702–6. 16208511 10.1007/s00330-005-2877-8

[23] Nelen MR Padberg GW Peeters EA Localization of the gene for Cowden disease to chromosome 10q22-23. Nat Genet 1996; 13: 114–6. 8673088 10.1038/ng0596-114

[24] Pilarski R Stephens JA Noss R Predicting PTEN mutations: an evaluation of Cowden syndrome and Bannayan-Riley-Ruvalcaba syndrome clinical features. J Med Genet 2011; 48: 505–12. 21659347 10.1136/jmg.2011.088807

[25] Stambolic V Suzuki A de la Pompa JL Negative regulation of PKB/Akt-dependent cell survival by the tumor suppressor PTEN. Cell 1998; 95: 29–39. 9778245 10.1016/s0092-8674(00)81780-8

[26] Williams AD LaRoy J Le T Clinical challenges in breast care for patients with PTEN pathogenic variants: a case series and literature review. Surg Oncol Insight 2024; 1: 100016.

[27] Riegert-Johnson DL Gleeson FC Roberts M Cancer and Lhermitte-Duclos disease are common in Cowden syndrome patients. Hered Cancer Clin Pract 2010; 8: 6. 20565722 10.1186/1897-4287-8-6 PMC2904729

[28] NCCN clinical practice guidelines in oncology. Genetic/familial high-risk assessment: breast, ovarian, and pancreatic, version 3. 2024, 2024. https://www.nccn.org/guidelines/guidelines-detail?category=2&id=1503. 10.6004/jnccn.2021.0001 33406487

